# Evaluation of statistical process control charts for infant mortality monitoring in Brazilian cities with different population sizes

**DOI:** 10.1186/s13104-024-06943-0

**Published:** 2024-10-08

**Authors:** Jéssica Souza, Cristiano Boccolini, Lais Baroni, Kele Belloze, Eduardo Bezerra, Marcel Pedroso, Ronaldo Fernandes Santos Alves, Eduardo Ogasawara

**Affiliations:** 1 Federal Center for Technological Education of Rio de Janeiro, CEFET/RJ, Rio de Janeiro, Brazil; 2 Institute of Scientific and Technological Communication and Information in Health, Oswaldo Cruz Foundation, Fiocruz, Rio de Janeiro, Brazil

**Keywords:** Brazil, Infant mortality, Diagnosis of health situation, Statistical process control, Control chart, Routinely collected health data

## Abstract

**Objectives:**

The control chart is a classic statistical technique in epidemiology for identifying trends, patterns, or alerts. One meaningful use is monitoring and tracking Infant Mortality Rates, which is a priority both domestically and for the World Health Organization, as it reflects the effectiveness of public policies and the progress of nations. This study aims to evaluate the applicability and performance of this technique in Brazilian cities with different population sizes using infant mortality data.

**Results:**

In this article, we evaluate the effectiveness of the statistical process control chart in the context of Brazilian cities. We present three categories of city groups, divided based on population size and classified according to the quality of the analyses when subjected to the control method: consistent, interpretable, and inconsistent. In cities with a large population, the data in these contexts show a lower noise level and reliable results. However, in intermediate and small-sized cities, the technique becomes limited in detecting deviations from expected behaviors, resulting in reduced reliability of the generated patterns and alerts.

## Introduction

Health situation analysis plays a crucial role in understanding the health status of populations and identifying their priority needs [1]. Given Brazil’s demographic, geographic, and socioeconomic diversity, cities are markedly different. Identifying these specificities and needs becomes paramount to ensure equitable healthcare for the population, regardless of their region of residence [2].

Indicators are measures usually adopted to monitor the health system throughout time. Due to their temporal patterns, time series are a powerful tool in epidemiology [3]. They are used in studies to forecast epidemic outbreaks [4], analyze disease trends or seasonality [5] and monitor and identify anomalies [6]. The statistical process Control Chart (CC) is a classic technique to establish acceptable values for indicators. This technique represents a widely employed statistical tool in quality management, control of epidemiological processes, and decision support. It enables monitoring of possible deviations, anomalies, trends, and seasonality. Unfitted values sign the need for specific interventions to improve the indicators for this population [7].

Among the various indicators that measure the quality of life of a population, Infant Mortality Rate (IMR) is one of the national and international priorities. This indicator estimates the risk of death of a child in their first year of life per thousand live births. In addition to reflecting the socioeconomic development of a country, the IMR highlights access to health for children and their families, as well as the effectiveness of interventions and public policies [8]. It plays a crucial role as an essential alert for epidemics, disease outbreaks, or humanitarian emergencies.

The CC [6] applied to IMRs enables identifying areas with higher vulnerability and inequalities, contributing to formulating more targeted and practical strategies to improve population health. This method determines the moment of action, generating an alert in the face of behavioral changes. Moreover, it enables a preventive approach focused on promoting maternal and child health and implementing measures to improve the quality of life for children, thus guaranteeing a healthier future for the entire society [9].

When considering the principles of universality and equity within Brazil’s Unified Health System (SUS), it is imperative to prioritize equal access to healthcare services for all cities and effective care for vulnerable populations [10]. This approach is essential to ensure that every community receives fair and equitable healthcare. Our study aims to evaluate the applicability and performance of the statistical process CC across Brazilian cities with different population sizes, utilizing IMR as the critical metric.

## Methods

The time series analysis used the infant mortality dataset from two major studies [11–13]. These datasets hold interoperable data on disease incidence, hospitalization, primary health care, vaccination, and breastfeeding [13]. The dataset gathers official and public information from the Live Birth Information System (SINASC) and the Mortality Information System (SIM) of the Brazilian Ministry of Health. Data extraction and processing occurred in August 2023. It included 31,416 infant deaths and 2,730,050 live births from 2009 to 2020.

They were temporal aggregated by municipalities according to months. Equation 1 describes the aggregation where $$y_j$$ is the aggregated observations computed as the mean of the original *m* observations associated with *j*. This aggregation approach enables summarizing daily observations and analyzing data on a broader temporal scale, such as weekly ($$m = 7)$$ or monthly ($$m = 30$$), facilitating the identification of long-term trends and significant patterns.

1 $$\begin{aligned} y_j = \sum _{t=m(j-1)+1}^{m.j} \frac{x_t}{m} \end{aligned}$$

The IMR is our selected indicator, calculated by the number of deaths of children under 1 year per 1,000 live births. Thus, the CC was built for all cities. It is necessary to remove epidemic periods to build the CC. While assuming a normal distribution, four steps are applied:

1. Definition of the study year;
2. Calculation of the monthly (or weekly) average (*MD*) for the ten years before the study year;
3. Calculation of the monthly (or weekly) standard deviation (*SD*) for the ten years before the study year;
4. Calculation of the upper and lower control limits. Typical observations fall between $[ \bar{x} - 1.96 \sigma , \bar{x} + 1.96 \sigma ]$, representing $95\%$ of the values in a normal distribution [14].

With all the values at hand, one can represent the graph and analyze the behavior of the observed values (study year) about the expected values and calculated limits.

The Brazilian cities were grouped into six population size categories according to the official classification of the Brazilian Institute of Geography and Statistics (IBGE) [15]. This classification helps to measure the performance of the CC for the groups. Each group can be classified into one of three categories presented in the results section: consistent, subject to interpretation, and inconsistent.

We automated all data processing and analysis using the free and open software R. The codes can be audited, replicated, and reused for alternative analysis. The proportion of missing data is $<0.01\%$.

## Results

The IBGE classifies Brazilian cities into six groups, varying according to population size (Table 1). It shows the total population (Tot. pop.), number of cities, live births, infant deaths, and the IMR (both mean and standard deviation) for each group. Due to differences in size and socioeconomic challenges in Brazil, each group has different characteristics, such as hospital and primary care structures.

**Table 1:** Profile of the Brazilian cities in 2020

Population size groups	Tot. Pop.	Cities	Live births	Infant deaths	IMR ($MD \pm SD$)	Classification
10,000 or less	12,733,775 (6%)	2,449 (44%)	150,928 (5.5%)	1,746 (5.6%)	10.8 ± 15.4	Inconsistent
10,001 to 20,000	19,023,018 (9%)	1,332 (24%)	240,720 (8.8%)	3,003 (9.6%)	12.3 ± 9.1	Interpretable/inconsistent
20,001 to 50,000	33,869,025 (16%)	1,112 (20%)	454,977 (16.7%)	5,646 (18%)	12.3 ± 6.7	Interpretable
50,001 to 100,000	24,150,300 (11.4%)	351 (6%)	332,265 (12.3%)	3,699 (11.8%)	11.0 ± 4,7	Consistent/interpretable
100,001 to 500,000	54,457,315 (25.7%)	277 (5%)	731,937 (26.8%)	8,039 (25.6%)	10.9 ± 3,1	Consistent
500,000 or more	67,522,259 (31.9%)	49 (1%)	819,223 (30%)	9,283 (29.5%)	11.6 ± 3.1	Consistent

For small cities, it is possible to observe more heterogeneity for IMR, where the *SD* equals 15.4. Generally, cities fall below the World Health Organization (WHO) target (12 per thousand live births [16]), while some are far from it. On the other hand, we find a reasonably homogeneous group when analyzing the IMR for the 49 largest cities in the country. Its mortality rate has an *SD* of 3.1, indicating that most cities have IMRs close to the *MD* of 11.6.

In this way, the *SD* of IMR proxies the applicability of the analysis of CC for the group of cities. The classification column of Table 1 represents it as consistent, interpretable, and inconsistent. Larger cities enable consistent adoption of CC. The method produces consistent results for some groups where the literature traditionally applies them, as seen in [17]. Conversely, smaller cities do not enable the direct adoption of CC. To this extent, the group was classified as inconsistent. It is critical since 44% of Brazilian cities are present in this group. Other groups were classified as interpretable with a certain degree between consistent and inconsistent according to the standard deviation of IMR. Interpretable are cities that might use CC with certain visual inspections to confirm problems.

### Consistent group

**Fig. 1:**
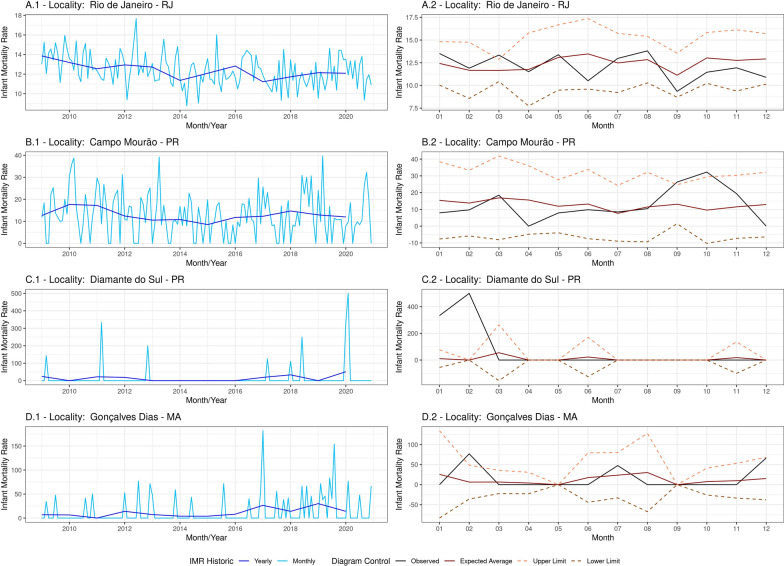
Historical Trend and CC of IMR for four Brazilian Municipalities. - Observed year: 2020—Limits (*SD*) and average (*MD*): 2009–2019

In this scenario, there is a clear indication of great utilization of the CC methodology. This behavior is observed in large cities with more than 100,000 inhabitants, such as Rio de Janeiro - RJ. Historically, this city has a large population volume, which generates data stability. With consistent data, in Fig. 1(A1), it is possible to analyze and identify patterns, such as the decrease in IMR from 14 per thousand live births to 12 per thousand live births, through the annual *MD*. There is also high oscillation in the monthly data over time. In Fig. 1(A2), by applying the CC, it is possible to identify the months with the highest and lowest mortality rates through the expected value—month six had the highest mortality, and month nine had the lowest. Additionally, the established limits for the city were determined. Interestingly, month three, which had the lowest degree of available oscillation, was precisely the month that showed an anomalous behavior when the observed value exceeded the upper expected limit. This point requires action or investigation by the competent authorities.

### Interpretable group

This group comprises situations where the results indicate warning signs, but their interpretation enables various analyses. Typically, this group includes cities of intermediate population size, such as Campo Mourão—PR. Due to population characteristics, there is a significant fluctuation in mortality data over the years. Although annual data remains stable, monthly data fluctuates between 0 and 40 per thousand live births, as seen in Fig. 1(B1). In the control analysis, in Fig. 1(B2), points where the observed value exceeds the upper limit may not necessarily represent a real alert, but they raise doubts. If there was a lower number of births in that month while the number of infant deaths remained constant, it is possible to have a false positive. This diagnosis requires an additional analysis of the absolute numbers and an understanding of whether there are other anomalies.

### Inconsistent group

The inconsistent group is observed in cities with medium and low population volumes, such as Diamante do Sul - PR and Gonçalves Dias - RS. Two behaviors fall into this category. Firstly, there are cities with minimal infant mortality history, with one or two cases per year, as exemplified in Fig. 1(C1). In these locations, the absence of a historical average and predefined limits generates inconsistency, as there is no basis for comparison. Figure 1(C2) exemplifies this phenomenon because this city had no infant deaths for several years. When deaths do occur, the numbers reach incredibly high levels, leading to inconsistent alerts and extremely high mortality rates. The alert in month two in Fig. 1(C2) was generated by one infant death out of two for the year.

Secondly, cities with available mortality history but with measured values are highly susceptible to rapid fluctuations, as shown in Fig. 1(D1). The monthly IMR oscillates between 0 and 130 deaths per thousand live births. In practice, a single additional or fewer births or infant deaths can cause the mortality rate to exhibit such behavior. In the case of Gonçalves Dias in Fig. 1(D2), it is possible to identify the trend of months with the highest and lowest historical volume of infant deaths, despite the IMR values assuming values outside the expected range (caused by monthly fluctuations). The alert for month two in Fig. 1(D2) is caused by two infant deaths out of three for that year. Once again, this demonstrates the vulnerability of the method to changes in absolute numbers.

## Discussions

Understanding past patterns and behaviors is crucial in epidemiological studies, including the analysis of infant mortality. However, when applying the CC technique to municipal scenarios, reliable results are only achieved for large cities with more stable data, representing just 6% of all municipalities, as shown in Table 1. The remaining 94% of municipalities face challenges with partially interpretable or inconsistent results. This difficulty arises from identifying value combinations that lead to changes in classification.

Traditionally, alternative approaches such as spatial or temporal aggregation have been used to overcome these issues. The spatial collection provides a comprehensive view of trends at state or macro-geographical levels but fails to capture intrinsic city characteristics [17]. Temporal aggregation examines data over quarters, semesters, or years, enabling the analysis of changes over time but losing the opportunity to address specific events in individual municipalities [7].

Discovering an adequate and balanced approach to tackling this complexity is crucial to ensure fair and unbiased analysis for all cities, regardless of their population size. By breaking the current invisibility in which many of these cities are submerged, we contribute to preserving the SUS principles and promoting equal treatment among locations. It is particularly relevant in crises, where the prompt action of competent authorities plays a fundamental role in containing extreme situations. Moreover, considering that cities with up to 20,000 inhabitants mainly obtain inconsistent results when subjected to the technique, as shown in Table 1, they represent 68% of the Brazilian municipalities. In other words, more than half of the country’s cities are subject to inconsistent analysis of infant mortality or other important indicators for societal development (ODS 3 [18]), such as tuberculosis and malaria, due to the inconsistency of the method in smaller cities.

While few studies use the CC technique for epidemic analysis at the municipal level [19], efforts should be made to adapt and improve the method for low population volumes and high data fluctuations. Alternative approaches like automated model selection for epidemic analysis [20] and Bayesian analysis for low-granularity area data [21] present possibilities but require further testing. This article reveals the potential for techniques to address the gaps presented.

A thorough exploration of data and methods can enhance understanding of IMRs in diverse cities, enabling proactive and effective analytical efforts to promote health and well-being in vulnerable communities. However, this paper aims to shed light on the need for a balanced approach to analyzing infant mortality, considering the limitations of the CC technique in smaller cities. It is crucial for fair and unbiased analysis and proactive health improvement initiatives in vulnerable communities.

## Limitations

- The SINASC and SIM, used to collect live births and deaths data, have variable coverages over time and across geographic units - i.e., lower at the beginning of historical series and underserved areas. Nevertheless, overall SINASC and SIM coverages are high at 98% and 96%, respectively.
- The method does not consider the growth, stability, or decline trend.
- The epidemic channel limits are dependent on the time window.

## Data Availability

The data described in this Research Note are freely and openly available on the Synapse repository at: (https://doi.org/10.7303/syn26454542). Anyone can browse the content on the Synapse website. However, the reader must register for an account using an email address to download the files and datasets. The R codes of the data analysis are available at: (https://github.com/cefet-rj-dal-members/ccimm).
